# Functional manganese ferrite/graphene oxide nanocomposites: effects of graphene oxide on the adsorption mechanisms of organic MB dye and inorganic As(v) ions from aqueous solution

**DOI:** 10.1039/c8ra00270c

**Published:** 2018-04-03

**Authors:** Pham Thi Lan Huong, Nguyen Tu, Hoang Lan, Le Hong Thang, Nguyen Van Quy, Pham Anh Tuan, Ngo Xuan Dinh, Vu Ngoc Phan, Anh-Tuan Le

**Affiliations:** Department of Nanoscience and Nanotechnology, Advanced Institute for Science and Technology (AIST), Hanoi University of Science and Technology (HUST) No. 1, Dai Co Viet Street, Hai Ba Trung District Hanoi Vietnam tuan.leanh1@hust.edu.vn + 84 4 23623 0293 + 84 4 23623 0435; School of Materials Science and Engineering, Hanoi University of Science and Technology Hanoi 10000 Vietnam; International Training Institute for Materials Science (ITIMS), Hanoi University of Science and Technology Hanoi 10000 Vietnam; Vietnam Metrology Institute 08 Hoang Quoc Viet Road, Cau Giay District Hanoi Vietnam; University of Transport Technology Hanoi 10000 Vietnam; Saigon University (SGU) 273 An Duong Vuong Street, Ward 3, District 5 Ho Chi Minh City Vietnam

## Abstract

In this study, manganese ferrite-graphene oxide (MFO-GO) nanocomposites were prepared *via* a co-precipitation reaction of Fe^3+^ and Mn^2+^ ions in a GO suspension. The effects of graphene oxide on the physicochemical characteristics, magnetic properties and adsorption activities of the MFO-GO nanocomposites were studied. Methylene blue (MB) and arsenic(v) were used in this study as model water pollutants. With an increase in the GO content in the range of 10 wt% to 50 wt%, the removal efficiency for both MB dye and arsenic(v) ions was improved. Our adsorption data revealed that the adsorption behavior of MB dye showed good agreement with the Langmuir isotherm model and pseudo-second-order equation, whereas the Freundlich isotherm model was more suitable for simulating the adsorption process of arsenic(v) ions on the MFO-GO nanocomposites. In addition, an important role of the GO content in the adsorption mechanisms of both MB dye and arsenic(v) ions was found, in which GO nanosheets play a key role in the mechanisms of electrostatic/ionic interactions, oxygen-containing groups and π–π conjugation in the case of the adsorption of MB dye, whereas the role of the GO content is mainly related to the mechanism of electrostatic/ionic interactions in the case of the adsorption of arsenic(v). Graphene oxide has the functions of increasing the number of active binding sites comprising oxygen-containing functional groups, reducing the agglomeration of MFO nanoparticles, increasing the number of adsorption sites, and improving the electrostatic/ionic interactions between adsorbents and adsorbates in order to enhance the adsorption performance of cationic organic dyes and/or heavy metal anions from aqueous solutions.

## Abstract 2

In this study, manganese ferrite-graphene oxide (MFO-GO) nanocomposites were prepared *via* a co-precipitation reaction of Fe^3+^ and Mn^2+^ ions in a GO suspension.

## Graphical abstract

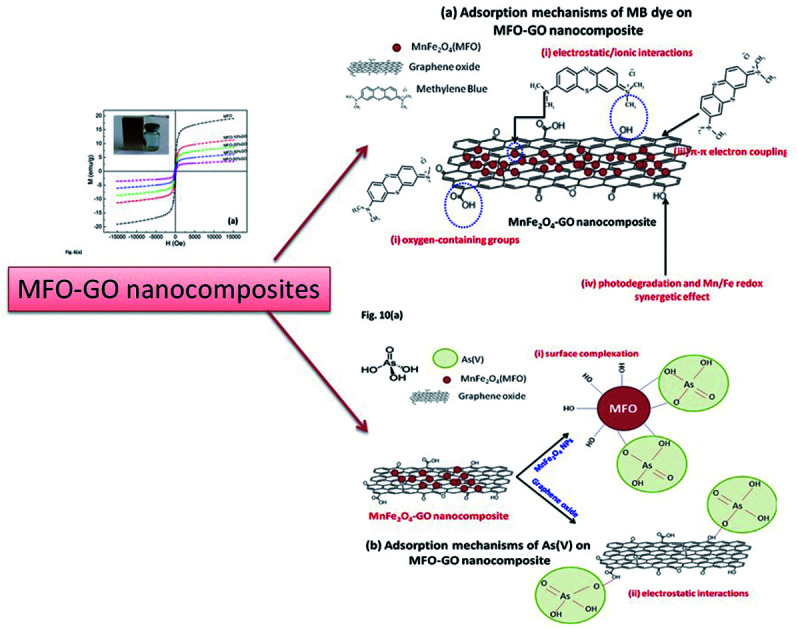

## 1. Introduction

Water is one of the most important substances on the earth because it is essential for life. The development of industrialization, civilization, increases in population and the uncontrolled use of agricultural chemicals have led to a decline in the quality of water resources.^1^ Currently, water pollution is a global problem and poses challenges in many developing countries owing to the shortage of suitable water treatment technologies. Water contaminants can be divided into different types such as inorganic pollutants (*e.g.*, heavy metals, arsenic, cadmium, chromium, and selenium), organic pollutants (*e.g.*, organic dyes, pesticides, and hydrocarbons) and biological pollutants (viruses and bacteria). Most of these pollutants are the main causes of outbreaks of infectious and non-communicable diseases, which are very dangerous for community health and ecosystems.^2^

Inorganic arsenic is a typical inorganic pollutant in water and has high toxicity and carcinogenicity. Contamination of groundwater with arsenic at high levels has been found and reported in some areas such as southeastern Asia, North and South America, and some countries in Europe.^3^ Long-term exposure to arsenic has been associated with different types of cancer and/or digestive problems. In order to lessen the dangerous effects of arsenic on human and community health, the World Health Organization (WHO) guideline level for arsenic in drinking water is about 10 ppb.

On the other hand, organic pollutants such as dyes, pesticides, fertilizers, hydrocarbons, and phenols have also been found in many water resources. It has been reported that the major causes of dye pollution result from the activities of industries such as textiles, paper, rubber, plastics, paints, printing, and leather. Dye pollutants can be divided into different types such as natural dyes, synthetic dyes and food dyes. Owing to the high toxicity of dyes and the discharge of such pollutants into water sources, dye pollution can lead to skin diseases and respiratory problems.^4^

Consequently, there is an imperative need to remove both inorganic and organic pollutant compounds from water to ensure healthy and safe water resources for people and communities. Currently, there are various technological methods for water treatment such as screening, filtration, centrifugal separation, sedimentation, coagulation, adsorption, and/or electrolysis. Among these techniques, adsorption methods are among the most promising solutions for water decontamination because of their low costs, high efficiency and flexibility in operation and the scale of treatment.^5^ There have been many intensive studies of the development of various nanomaterial-based adsorbents such as graphene, carbon nanotubes, metal oxide/magnetic nanoparticles, and their composites/hybrids for the removal of pollutants from water.^6–9^ Among existing nanoadsorbents, manganese ferrite-graphene oxide (MFO-GO; MFO = MnFe_2_O_4_) nanocomposites^10–12^ have attracted much interest for the highly efficient removal of both organic dyes, *e.g.*, methylene blue (MB), and heavy metal ions, *e.g.*, arsenic(iii), arsenic(v), and lead(ii). For example, Fu *et al.*^10^ reported that the combination of MFO nanoparticles with graphene sheets led to high photoelectrocatalytic activity in the degradation of MB under visible-light irradiation in the absence of hydrogen peroxide, although the MFO nanoparticles alone were photocatalytically inactive. The removal efficiency was found to be 96.2% after 360 min. More recently, Peng *et al.*^11^ also reported the use of rGO-MFO catalysts for the efficient decomposition of MB dye in the presence of H_2_O_2_. They showed that MB dye molecules in a neutral solution can be fully decomposed within 130 min in the presence of H_2_O_2_ at room temperature. In another work, Kumar *et al.*^12^ showed that hybrids of single-layer graphene oxide with MFO nanoparticles exhibited the best adsorption properties for the efficient removal of lead(ii), arsenic(iii), and arsenic(v) from contaminated water. They also revealed that the adsorption capacities of the GO-MFO nanohybrids were superior to those of all reported adsorbents for the removal of lead(ii), arsenic(iii), and arsenic(v). The maximum adsorption capacities of the GO-MFO nanohybrids reached values of 673 mg g^−1^ for lead(ii), 146 mg g^−1^ for arsenic(iii) and 207 mg g^−1^ for arsenic(v). All the reported studies showed that the GO-MFO composite systems exhibited better adsorption/catalysis activity than that of pure MFO nanoparticles. In addition, some possible mechanisms of the enhancement in the catalysis/adsorption activity of the GO-MFO systems were mentioned: (i) firstly, the significant enhancement in photoactivity toward MB molecules could be ascribed to the reduction of graphene oxide, because the excellent conductivity of reduced GO sheets was favorable for the efficient separation of photogenerated carriers in the coupled MFO/graphene system; (ii) secondly, the high catalytic performance of the rGO/MFO hybrid toward MB dye is not only attributed to the synergetic effects of rGO and MFO but is also related to redox couples of Fe/Mn ions during the reaction; and (iii) thirdly, the improved adsorption property for the heavy metal ions lead(ii), arsenic(iii), and arsenic(v) was due to the combination of the unique layered nature (which maximized the surface area) of the hybrid system and the high adsorption capabilities of both GO and MFO nanoparticles. Although the above mentioned explanations provide an understanding of the mechanism of the enhancement in the catalytic/adsorption activity of the GO-MFO systems, the effect of the GO content on the adsorption/catalytic properties of GO-MFO systems has, however, not been fully examined. To the best of our knowledge, there has been no systematic study of the effects of the GO content on the adsorption activity of both the organic dye MB and inorganic arsenic ions. Thus, a full understanding of the influence of the GO content on the mechanisms of the adsorption of pollutants from aqueous solutions is still a challenge that requires further investigation.

In the present study, we investigated the effects of the GO concentration on adsorption activities and the corresponding adsorption mechanisms. GO-MFO nanocomposites with different GO contents in the range of 10–50 wt% were synthesized using a modified co-precipitation method. We employed the prepared MFO-GO nanocomposites for studies of the adsorption of the organic dye MB and inorganic arsenic(v) ions from aqueous solutions, and their adsorption activities were compared with those of pure MFO nanoparticles and GO nanosheets. The adsorption kinetics of the organic dye MB and inorganic arsenic(v) ions on the GO-MFO nanocomposites was also studied in detail. Finally, beneficial effects of the GO content on the adsorption mechanisms of magnetically separable GO-MFO adsorbents were proposed.

## 2. Experimental procedures

### 2.1. Chemicals

Analytical-grade manganese chloride tetrahydrate (MnCl_2_·4H_2_O, ≥99%), ferric chloride hexahydrate (FeCl_3_·6H_2_O, ≥99%), sodium hydroxide (NaOH), ammonium hydroxide (NH_4_OH, 25%), potassium permanganate (KMnO_4_, 99.9%), hydrogen peroxide (H_2_O_2_, 30%), sulfuric acid (H_2_SO_4_, 98%), hydrochloric acid (HCl, 37%), and nitric acid (HNO_3_, 63%) used in this study were purchased from Shanghai Chemical Reagent Co. Ltd. Methylene blue (MB, C_16_H_18_ClN_3_S·3H_2_O) and arsenic(v) used as model pollutant agents were obtained from HanChem Chemical Agent Company (Daejeon, Korea).

### 2.2. Synthesis of MnFe_2_O_4_-GO (MFO-GO) nanocomposites

Pure GO nanosheets were synthesized from coal powder by the modified Hummers' method, whereas bare MFO nanoparticles were synthesized by a co-precipitation method. Details of the synthesis procedures can be found elsewhere.^13,14^ The pure MFO nanoparticles and GO nanosheets were used for purposes of comparison with the MFO-GO nanocomposites.

Similarly, MFO-GO nanocomposites were synthesized by a modified co-precipitation method. FeCl_3_·6H_2_O and MnCl_2_·4H_2_O were dissolved in deionized water with a molar ratio of Mn : Fe in the solution of 1 : 2. The resulting mixture was mixed with various suspensions of GO nanosheets in the range of 10–50 wt% under stirring for 30 min. The mixed solution was then constantly stirred and heated to 80 °C. Next, 20 mL of a 0.5 M NaOH solution was added slowly to the solution of the complex. The color of the solution changed immediately from orange to dark brown after the addition of NaOH, which indicated the formation of MFO nanoparticles. The reaction mixture was then kept at a temperature of about 80 °C for 1 h. The product, namely, the MFO-GO nanocomposite, was separated from the solution by an external magnetic field and washed several times with deionized water and acetone.

### 2.3. Characterization techniques

Transmission electron microscopy (TEM, JEOL JEM-1010) was conducted to determine the morphology of samples. In addition, high-resolution transmission electron microscopy (HRTEM, FEI Tecnai, 200 kV) was employed to determine the morphology and distribution of the MFO nanoparticles on the GO nanosheets. Samples for HRTEM characterization were prepared by placing a drop of a colloidal solution on a carbon-coated copper grid that was dried at room temperature. The composition of the MFO-GO nanocomposite was characterized by energy-dispersive X-ray analysis (JEOL 5410 LV). The crystalline microstructure of all samples that were prepared was analyzed by X-ray diffraction (XRD, Bruker D5005) using Cu Kα radiation (*λ* = 0.154 nm) in steps of 0.02° (2*θ*) at room temperature. The background was subtracted by the linear interpolation method. The chemical groups were analyzed using Fourier transform infrared (FTIR) spectroscopy measurements. Samples were collected, coated with one layer of potassium bromide and compressed into pellets, and spectra were recorded in the range from 400 cm^−1^ to 4000 cm^−1^ with a Nicolet 6700 FTIR instrument. Magnetization curves of MFO nanoparticles and MFO-GO nanocomposites were recorded with a vibrating sample magnetometer (VSM, MicroSense EV9) at room temperature. The chemical state of samples was studied by X-ray photoelectron spectroscopy (XPS). The Brunauer–Emmett–Teller (BET) surface area and pore diameter of samples were determined from the adsorption of N_2_ at 77 K using a Micromeritics ASAP 2000 system.

### 2.4. Arsenic(v) adsorption experiments

Batch experiments were conducted to study the adsorption behavior and kinetics of the adsorption of arsenic(v). A standard arsenic(v) solution (H_3_AsO_4_/HNO_3_ 0.5 M) was prepared with various concentrations from 0 to 50 mg L^−1^ for the generation of calibration curves for the determination of arsenic. The concentration of arsenic(v) was measured using atomic adsorption spectroscopy (AAS) in accordance with standard methods. The amount of the adsorbent material (GO, MFO, or MFO-10–50 wt% GO) used for each experiment was fixed at 0.02 g. The volume of the tested arsenic(v) solution was 100 mL.

Firstly, the initial concentration of arsenic(v) was fixed at 30 mg L^−1^, and the pH was kept at 1–2. The adsorption behavior of samples (GO, MFO and MFO-GO) was investigated at various adsorption times from 10 to 90 min. Secondly, to understand the adsorption kinetics and determine the maximum adsorption capacities of the absorbents, arsenic(v) solutions of various concentrations ranging from 10 mg L^−1^ to 50 mg L^−1^ were prepared, and the equilibration time was fixed at 20 min, and the pH was kept at 1–2.

### 2.5. Methylene blue adsorption experiments

Adsorption experiments were performed using a batch method with a shaking speed of 200 rpm. Typically, 0.02 g of adsorbent material (GO, MFO, or MFO-10–50 wt% GO) was added to 100 mL of a solution containing various amounts of the cationic dye MB. The concentrations of MB in the solutions were determined by measuring the absorbance of the solutions at 660 nm with a UV-vis spectrophotometer at room temperature.

In these experiments, the initial concentration of the MB solution was fixed at 10^−5^ mol L^−1^, the pH was 7, and the adsorption behavior of samples (GO, MFO and MFO-GO) was studied at various adsorption times from 5 to 90 min. MB solutions with various concentrations ranging from 7 × 10^−6^ mol L^−1^ to 25 × 10^−6^ mol L^−1^ were prepared, and the equilibration time was fixed at 20 minutes and the pH at 7 for the study of the adsorption kinetics and maximum adsorption capacity of the adsorbents. All measurements of the adsorption of MB and arsenic(v) were conducted at room temperature (25 °C).

The adsorption amount and adsorption rate (percentage removal) were calculated from the difference in the pollutant concentration in the aqueous solution before and after adsorption according to the following equations:^15^1
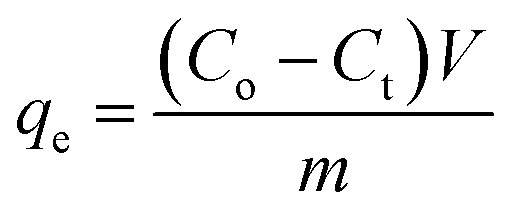
2
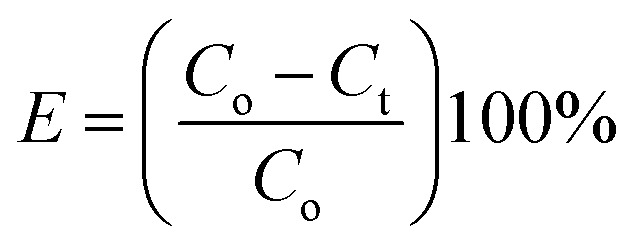
where *q*_e_ is the amount of pollutant (mg g^−1^) adsorbed on the adsorbent at equilibrium, *E* is the removal efficiency (%) of the adsorbent, *C*_o_ and *C*_t_ (mg L^−1^) are the initial pollutant concentration and the pollutant concentration at equilibrium, respectively, *V* (L) is the volume of the solution, and *m* (g) is the mass of the adsorbent. The two typical pollutants used in this study were methylene blue and arsenic(v).

## 3. Results and discussion

### 3.1. Morphology and microstructure analysis

We used a two-step process to synthesize MFO-GO magnetic nanocomposites for adsorption studies. The first step comprised producing GO nanosheets with oxygen-containing functional groups using a modified Hummers' method. In the second step, MFO nanoparticles were deposited on the surface of GO sheets *via* a co-precipitation reaction of Fe^3+^ and Mn^2+^ ions in a GO suspension to obtain water-dispersible MFO-GO nanocomposites. The formation of MFO nanoparticles on the surface of GO nanosheets was confirmed using TEM and XRD measurements. The fundamental reaction in the formation of MFO nanoparticles can be expressed as follows:

3 MnCl_2_ + 2FeCl_3_ + 8NaOH → MnFe_2_O_4_ + 8NaCl + 4H_2_O

**Fig. 1:**
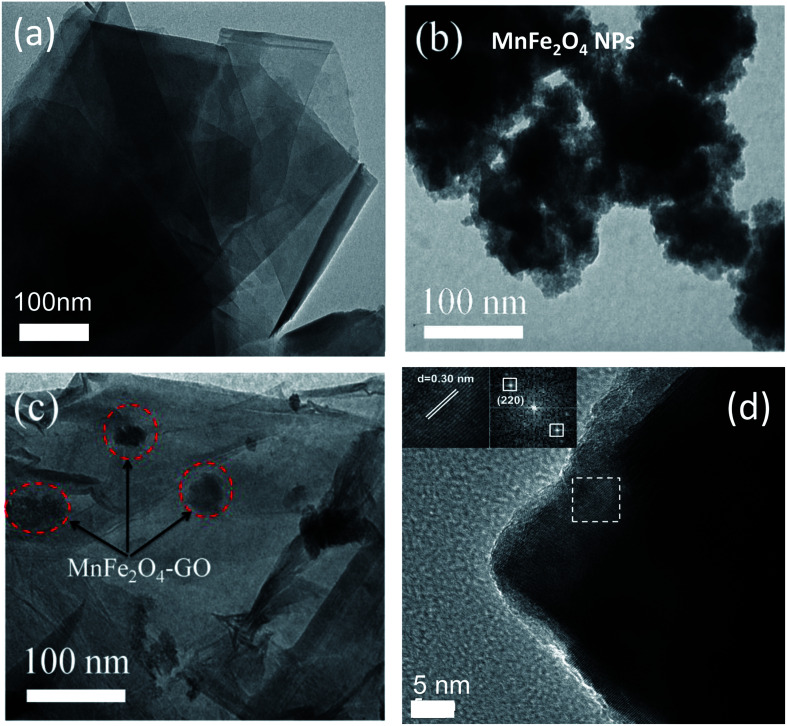
TEM images of (a) pure GO nanosheets, (b) bare MnFe_2_O_4_ nanoparticles, and (c) GO-MnFe_2_O_4_ nanocomposites and (d) HRTEM image of MnFe_2_O_4_ nanocrystals. The inset of (d) shows the lattice and FFT image of selected areas in the HRTEM image.

Firstly, TEM measurements were employed to study the morphology and shape of the studied adsorbent materials, namely, MFO nanoparticles, GO nanosheets and MFO-GO nanocomposites. Fig. 1 shows TEM images of (a) GO nanosheets, (b) MFO nanoparticles, and (c) MFO-GO nanocomposites and (d) an HRTEM image of MFO nanocrystals formed on GO nanosheets. It can be seen from Fig. 1(a) that the GO nanosheets were transparent. Besides, the presence of wrinkles was observed, which indicated that the GO sheets were thin.^16^ In contrast, as can be seen from Fig. 1(b), the MFO nanoparticles exhibited a nanocluster-shaped morphology. These nanoclusters displayed extensive agglomeration between particles. However, when MFO nanoparticles were deposited on GO nanosheets, the nanoclusters were well dispersed on the surface of the GO nanosheets [see Fig. 1(c)]. Our results suggest that the addition of GO sheets can help to prevent MFO nanoparticles from aggregating to form large MFO nanocrystals. The high-resolution TEM (HRTEM) image (see Fig. 1(d)) shows well-resolved lattice fringes of as-formed MFO nanocrystals. The lattice spacing was measured to be ∼0.30 nm, which corresponds to the (220) lattice plane of manganese ferrite. The TEM analysis revealed that after the precipitation reaction MFO nanoparticles were anchored to the surface of GO nanosheets.

**Fig. 2:**
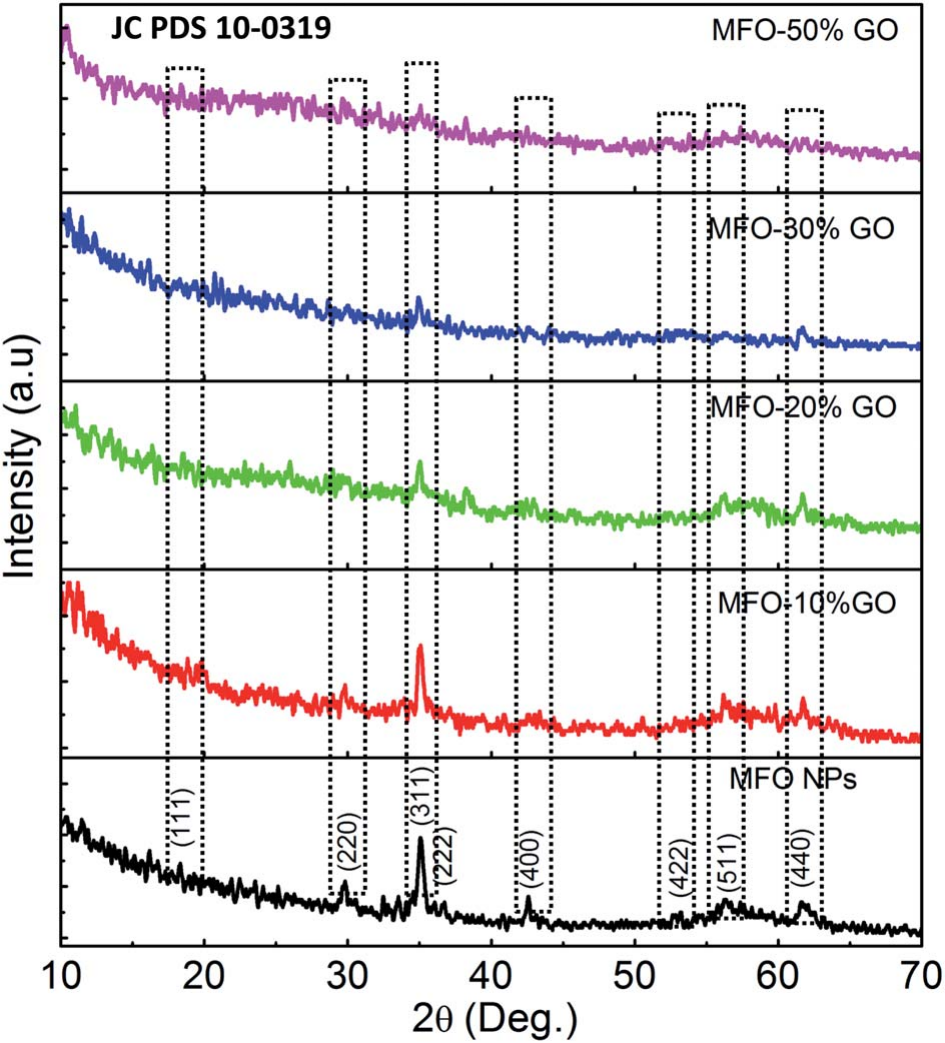
XRD patterns of bare MnFe_2_O_4_ nanoparticles and GO-MnFe_2_O_4_ nanocomposites prepared with various GO concentrations from 10 wt% to 50 wt%.

Secondly, XRD measurements were also employed to confirm the crystalline nature of MFO nanoparticles, as shown in Fig. 2. For bare MFO nanoparticles, the XRD pattern exhibits eight characteristic peaks at 2*θ* = 18.9°, 29.7°, 34.98°, 36.5°, 42.52°, 52.63°, 56.19° and 61.96°, which were indexed to the (111), (220), (311), (222), (400), (422), (511) and (440) planes, respectively. These peaks are similar to those in JCPDS 10-0319 for the cubic spinel ferrite structure of MnFe_2_O_4_. For MFO-GO nanocomposites similar peaks were also found, but their intensity decreased with an increase in the GO concentration. It can be seen that for the MFO-50% GO sample these diffraction peaks were fairly weak, and the structure of the sample was similar to that of the amorphous phase. These results suggest that an increase in the GO concentration in a sample of an MFO-GO nanocomposite has a strong effect on the connectivity and distribution of MFO nanoparticles on the surface of GO nanosheets.

### 3.2. Surface interaction analysis

**Fig. 3:**
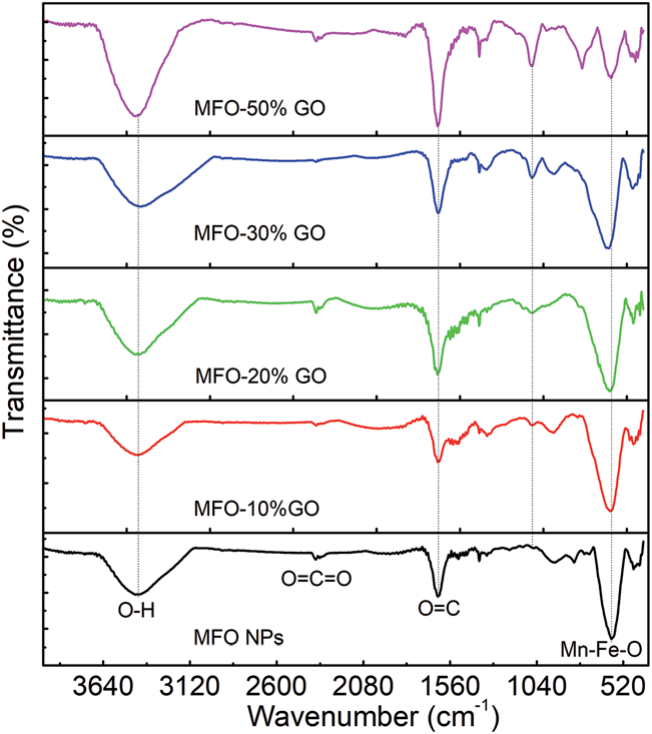
FTIR spectra of bare MnFe_2_O_4_ nanoparticles and GO-MnFe_2_O_4_ nanocomposites prepared with various GO concentrations from 10 wt% to 50 wt%.

To determine the effects of the GO concentration on the surface interactions of samples, we used FTIR measurements. Fig. 3 shows the FTIR spectra of MFO nanoparticles and samples of MFO-GO nanocomposites with various GO concentrations from 10 wt% to 50 wt%. For all the samples, a broad absorption band can be seen at 3450 cm^−1^, which corresponds to the normal polymeric O–H stretching vibrations of H_2_O. The band at 1635 cm^−1^ is associated with the stretching of the C=O bond in carboxyl groups, whereas the absorption peaks at 1337 cm^−1^ and 1057 cm^−1^ correspond to the stretching of epoxide groups, which indicates the existence of functional groups on the GO nanosheets. The absorption peak around 581–594 cm^−1^ is a characteristic peak corresponding to the stretching vibrations of the Mn–Fe–O linkage. Previous studies^11,12^ also indicated variations in the intensity of stretching absorption and slight shifts of the peak due to stretching vibrations of the Mn–Fe–O linkage in MFO-GO nanocomposites in comparison with MFO nanoparticles, which revealed that the MFO nanoparticles were bound to the GO surface *via* the formation of a coordinate bond or *via* simple electrostatic attraction between MFO nanoparticles and remaining functional groups on both the basal planes (hydroxyl groups (–OH)) and edges (carboxyl groups (–COOH) or epoxy groups (C–O–C)) of the GO sheets.

With an increase in the GO concentration, the intensity of the peaks due to stretching vibrations of functional groups increased, which was due to a large increase in the amounts of various functional groups such as hydroxyl, carboxyl and epoxide groups on GO nanosheets with an increase in the GO content. However, the intensity of the peak due to stretching vibrations of Mn–Fe–O linkages decreased when the MFO nanoparticles were bound by different layers of GO nanosheets. This result confirms that an increase in the GO concentration had an effect on the distribution of oxygen-containing functional groups in graphene oxide and therefore also an effect on the surface interactions of MFO nanoparticles with the remaining functional groups on GO nanosheets. This feature is important and will influence the adsorption activity of adsorbent materials; thus, the selection of a suitable GO/MFO ratio is necessary for optimization of the adsorption behavior of magnetic adsorbents.

### 3.3. Chemical state analysis

As mentioned above, an increase in the GO concentration had a strong influence on the distribution and variation of oxygen-containing functional groups in graphene oxide. To further confirm this effect, we employed XPS measurements for the analysis of the surface chemical bonding state of samples.

**Fig. 4:**
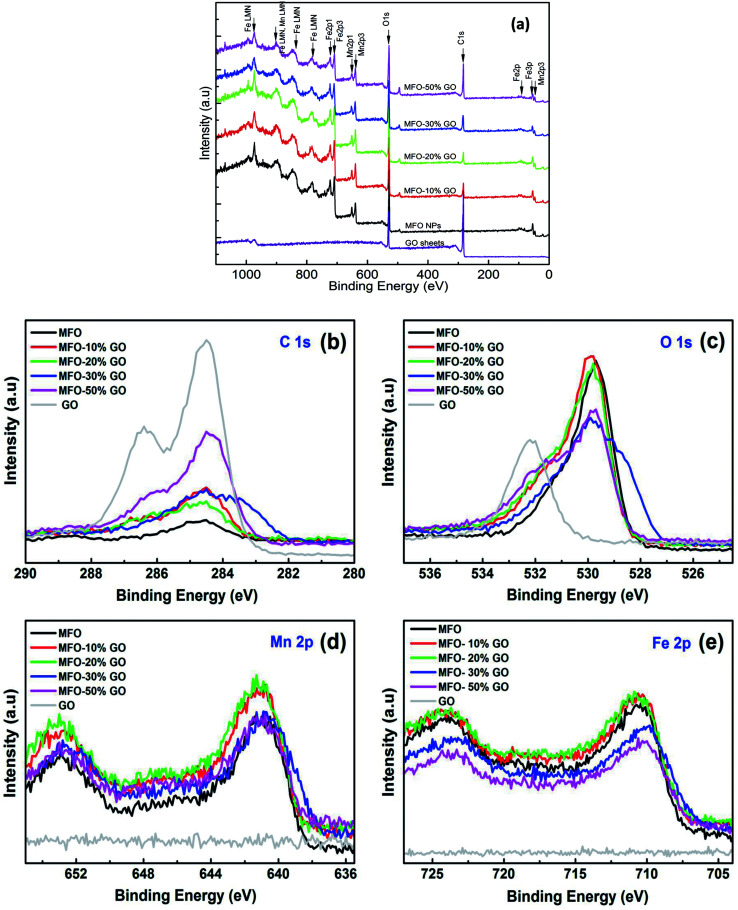
XPS analysis: (a) survey scan of all chemical elements, (b) C 1s spectra, (c) O 1s spectra, (d) Mn 2p spectra and (e) Fe 2p spectra of all studied samples.

**Fig. 5:**
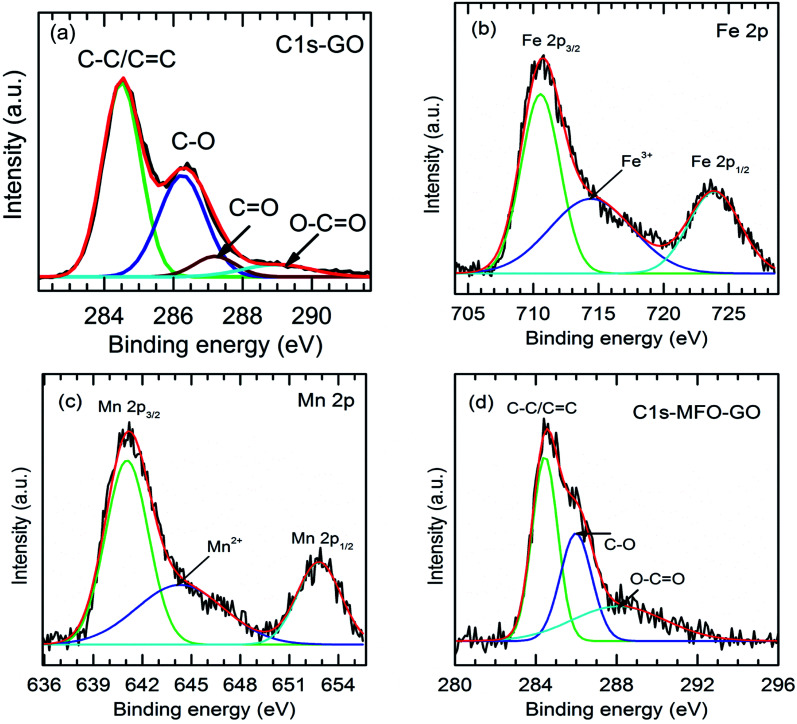
Fitted XPS analysis of (a) C 1s (GO sample); (b) Fe 2p, (c) Mn 2p and (d) C 1s for GO-MnFe_2_O_4_ nanocomposite (20 wt%).

Full-scale XPS survey spectra with C 1s, O 1s, Mn 2p, and Fe 2p peaks for all the studied samples are shown in Fig. 4(a). The detailed elemental spectra of C 1s, O 1s, Mn 2p, and Fe 2p are also displayed in Fig. 4(b–e), respectively. As can be seen in Fig. 5(a), for pure GO nanosheets the C 1s peaks at 284.5 eV, 286.5 eV, 287.2 eV and 288.9 eV are associated with sp^2^ C–C/C=C, C–O, C=O and O–C=O bonds, respectively, which indicates the presence of carbon element. However, in comparison for the MFO-GO nanocomposites the intensities of the peaks due to C–C/C=C and C–O bonds decreased drastically. With an increase in the GO concentration, the peak due to C–C/C=C bonds became predominant (284.5 eV), whereas the peak due to C–O bonds (286.5 eV) was reduced (see Fig. 4(b) and 5d). This reflects the fact that GO was reduced to rGO after the co-precipitation process, in which a small number of oxygen-containing groups were reduced. In the O 1s spectra (see Fig. 4(c)), the peaks at 529.5 eV and 530.8 eV correspond to the presence of O^2−^ ions and O–H bonds. With an increase in the GO concentration, the intensities of the peaks due to O^2−^ ions and O–H bonds increased drastically. This means that the number of oxygen-containing groups in the samples increased with an increase in the GO concentration. It is obvious that the XPS spectrum of Mn 2p (Fig. 5(c)) has two characteristic main peaks at binding energies of 641.2 eV (Mn 2p_3/2_) and 652.8 eV (Mn 2p_1/2_), together with a satellite peak at 644.6 eV, which indicates the presence of the Mn^2+^ chemical state within MnFe_2_O_4_ nanoparticles. It is also found that the XPS spectrum of Fe 2p (Fig. 5(b)) displays two main peaks at 711.2 eV (Fe 2p_3/2_) and 725.1 eV (Fe 2p_1/2_), and one satellite peak appears at 714.5 eV, which reflects the presence of the Fe^3+^ chemical state. These results that were obtained indicate that with an increase in the GO concentration the chemical states of oxygen-containing groups varied and the quantity of C–C/C=C bonds increased, whereas the chemical state of MFO nanoparticles was nearly unchanged (see Fig. 4(d and e) and 5(b and c)).

### 3.4. Magnetic analysis

**Fig. 6:**
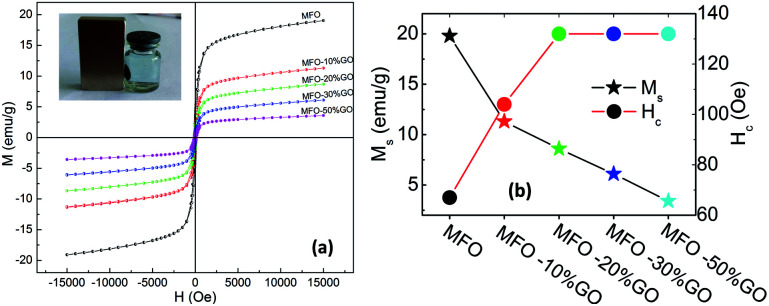
(a) Room temperature magnetic hysteresis (M–H) curves for bare MnFe_2_O_4_ nanoparticles and GO-MnFe_2_O_4_ nanocomposites and (b) the saturation magnetization (*M*_s_) and coercivity (*H*_c_) values determined from the magnetic hysteresis curves. The inset of (a) demonstrates the magnetic separation ability of MFO-GO sample by using a small magnet.

In order to investigate the effects of the GO concentration on the magnetic behavior of the studied samples, we used VSM measurements. Fig. 6(a) shows magnetization *versus* magnetic field (M–H) curves for bare MFO nanoparticles and samples of MFO-GO nanocomposites with various GO concentrations from 10 to 50 wt%. Magnetic parameters of all the studied samples were collected and are displayed in Fig. 6(b). The results showed that the saturation magnetization (*M*_s_) of the bare MFO nanoparticles was about 20 emu g^−1^, whereas the *M*_s_ values of the MFO-GO nanocomposite samples decreased with an increase in the GO concentration from 12.2 emu g^−1^ (10% GO content) to 3.2 emu g^−1^ (50% GO content). This was expected because the measurements were normalized by the total mass of the sample including both GO and MFO. We also found that the coercivity (*H*_c_) of the MFO-GO nanocomposite samples increased in comparison with that of the bare MFO nanoparticles. With an increase in the GO concentration, the *H*_c_ value of the MFO-GO nanocomposites increased from 100 Oe to 130 Oe (see Fig. 6(b)). This could be related to a decrease in the average interparticle distance with an increase in the GO content, which increased the interparticle interactions and therefore tended to increase the *H*_c_ value. Importantly, with their relatively high magnetization values, the samples of the MFO-GO nanocomposites can be easily removed from solutions and recycled by applying an external magnetic field (see inset in Fig. 6(a)). This property is very important for practical adsorbent materials in order to enhance their usage efficiency because it facilitates their reusability and magnetic separation.

### 3.5. Surface area analysis

**Fig. 7:**
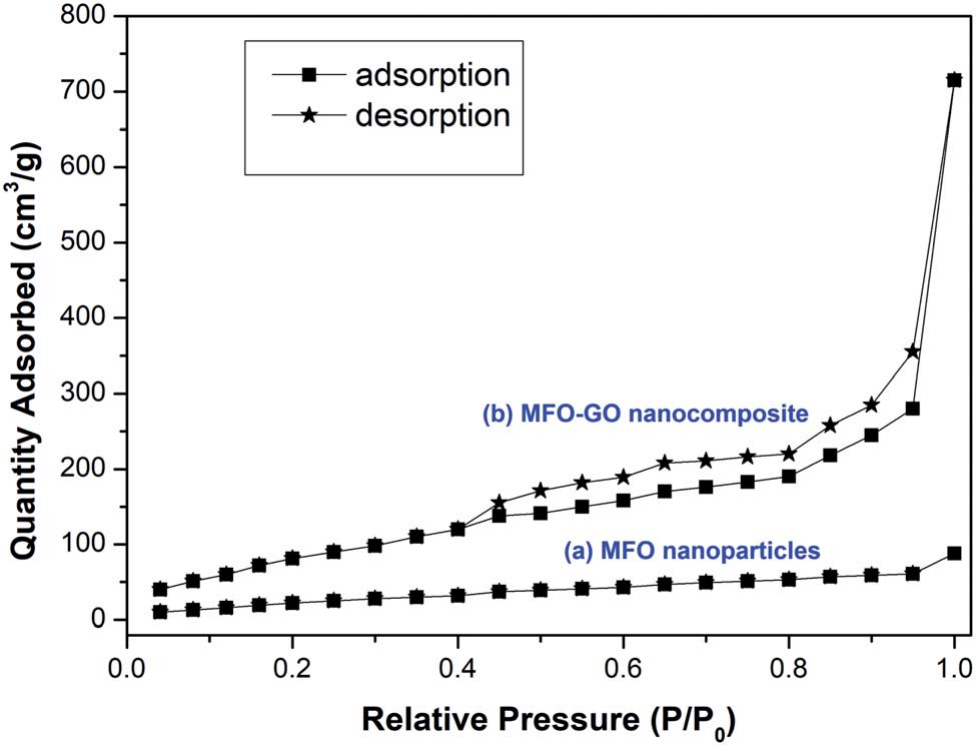
Nitrogen adsorption–desorption isotherms for (a) bare MFO nanoparticles and (b) the MFO-GO (20 wt%) nanocomposite.

The textural properties and nitrogen adsorption–desorption isotherms of MFO nanoparticles and an MFO-GO nanocomposite are shown in Fig. 7. The BET surface area of the MFO-GO nanocomposite was found to be about 316.8 m^2^ g^−1^, which was larger than that of the bare MFO nanoparticles of 67.5 m^2^ g^−1^. These surface area values are comparable to those reported elsewhere.^12,15,17^ It was revealed that the specific surface area of nanocomposite samples increased with an increase in the GO content, which was mainly derived from graphene oxide nanosheets. The Barrett–Joyner–Halenda (BJH) method was employed to calculate the total pore volume and average pore size of samples. The calculated results indicated that the total pore volume and corresponding average pore size of an MFO-GO nanocomposite were about 0.97 cm^3^ g^−1^ and 4.2 nm, respectively, whereas those of bare MFO nanoparticles were calculated to be about 0.18 cm^3^ g^−1^ and 5.6 nm, respectively. Our results suggest that the larger surface area and pore volume of the MFO-GO nanocomposite could provide more possible sites for adsorption and reactions and consequently lead to a higher adsorption capacity in comparison with that of bare MFO nanoparticles. It was also found that the nitrogen adsorption–desorption isotherm of the MFO-GO nanocomposite displays a type IV curve and H3 hysteresis loop according to the IUPAC (International Union of Pure and Applied Chemistry) classification.^17^ The average pore sizes were about 4.2–5.6 nm, which indicated the mesoporous nature of the nanomaterials,^12–18^ which is very promising for adsorption applications.

### 3.6. Adsorption analysis

Finally, to complete this study, the effects of the GO concentration on the adsorption activity, removal efficiency and adsorption mechanisms of MB dye and arsenic(v) in solution were examined.

#### a. Adsorption activity and removal efficiency

**Fig. 8:**
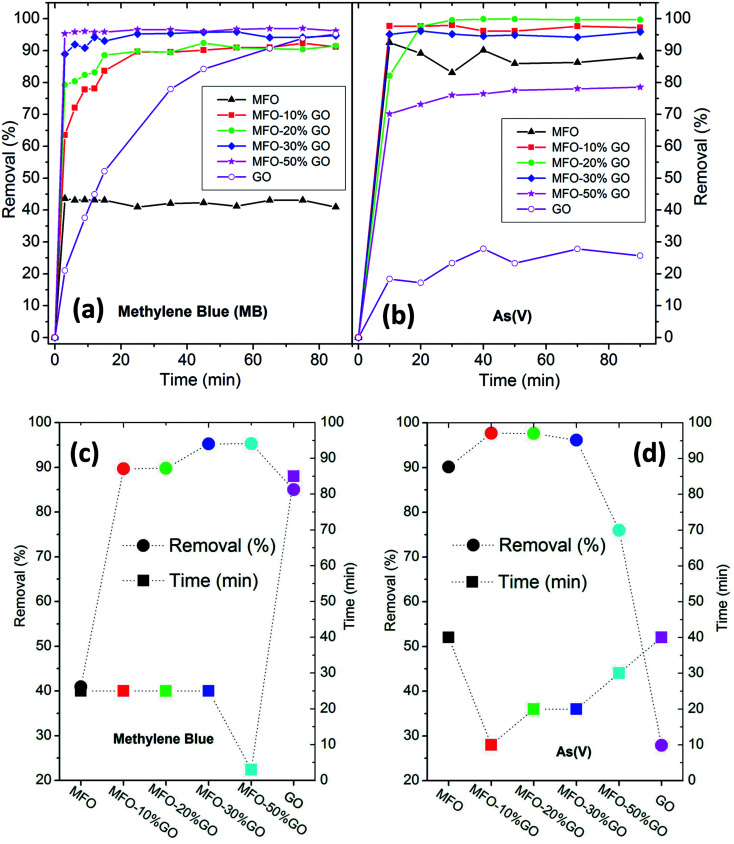
Changes in removal efficiency as a function of contact time for all investigated samples for the removal of (a) methylene blue (MB) dye and (b) arsenic(v) ions. The changes in removal efficiency and saturated adsorption time for all samples with (c) MB dye and (d) arsenic(v) ions.

The adsorption activity of various adsorbent materials, including MFO nanoparticles, GO nanosheets, and MFO-GO (10–50 wt%) nanocomposites, was investigated. MB dye and arsenic(v) ions were selected as cationic and anionic pollutants, respectively, for adsorption studies. In adsorption processes, the contact time of the adsorbent materials with pollutants is an important parameter for evaluation of the adsorption activity.^15–17^ In our present study, the adsorption process for all adsorbent samples was examined at different contact times from 5 min to 90 min. The calculated removal efficiency of all samples as a function of the adsorption time is shown in Fig. 8(a) for MB dye and Fig. 8(b) for arsenic(v) ions. It is noted that the measured concentrations of MB dye and arsenic(v), as well as the calculated removal efficiencies, are the average values for three experiments obtained from UV-vis and AAS measurements, respectively. The estimated errors in the UV-vis and AAS measurements were about 5%.

Firstly, in the case of the adsorption of MB, as shown in Fig. 8(a), it can be seen that bare MFO nanoparticles displayed a removal efficiency of ∼42%, whereas that of pure GO nanosheets was >80%. Moreover, all the MFO-GO nanocomposites (10–50 wt% GO content) exhibited higher removal efficiency than those of both pure MFO nanoparticles and GO nanosheets. It is also obvious that the removal efficiency for MB dye increased with an increase in the GO concentration from 91% for a sample of the MFO-10 wt% GO nanocomposite to 95% for a sample of the MFO-50 wt% GO nanocomposite. Furthermore, the saturation adsorption time for the MFO-GO nanocomposites (∼25 min) was much less than that for pure GO nanosheets (∼85 min). A different tendency was found in the case of the adsorption of arsenic(v), as shown in Fig. 8(b). It is obvious that pure GO nanosheets exhibited a removal efficiency of ∼27%, whereas that of bare MFO nanoparticles was about 90%. The removal efficiency of the MFO-GO nanocomposites increased with an increase in the GO concentration, reached a maximum of ∼99.9% for a sample of the MFO-20 wt% GO nanocomposite, and decreased drastically with a further increase in the GO concentration to ∼76% for a sample of the MFO-50 wt% GO nanocomposite. These results indicate the significant influence of the GO concentration on the adsorption activity and removal efficiency.

Important adsorption parameters, namely, the removal efficiency and saturation adsorption time, for all studied samples are displayed in Fig. 8(c and d). For MB adsorption activity, the removal efficiency of the MFO-GO nanocomposites was higher than those of both bare MFO nanoparticles and GO nanosheets, whereas their saturation adsorption time was similar to that of bare MFO nanoparticles and much shorter than that of pure GO nanosheets. For arsenic(v) adsorption activity, the saturation adsorption time of the MFO-GO nanocomposites was much less than those of both bare MFO nanoparticles and GO nanosheets. The removal efficiency of the MFO-GO nanocomposites was drastically altered by the adjustment of the GO concentration in the composite samples. These findings suggest that the selection of the GO concentration is important for optimization of the adsorption activity, including the removal efficiency and saturation adsorption time, for both cationic and anionic pollutants.^18,19^

#### b. Adsorption kinetics

In order to obtain a further understanding of the adsorption behavior of samples of the MFO-GO nanocomposites, we investigated the adsorption kinetics of the adsorbent materials. In an adsorption process, the determination of the adsorption kinetics is necessary for understanding the adsorption mechanism of the adsorbents.^20,21^ Quantification of the changes in sorption with time requires an appropriate kinetic model. In our present study, two established models, namely, the pseudo-first-order and pseudo-second-order models, were employed to understand the kinetics of the sorption process of MB dye and arsenic(v) by various adsorbents.

The pseudo-first-order equation can be expressed as:4
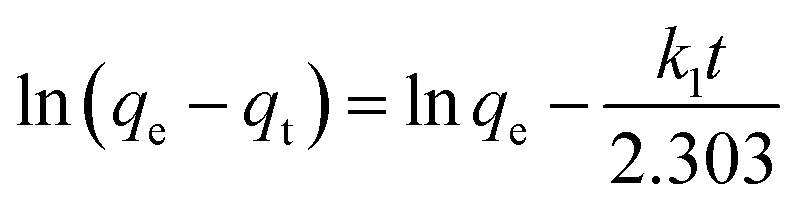


The pseudo-second-order equation can be expressed as:5
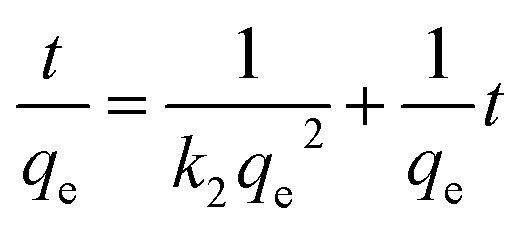
where *k*_1_ is the rate constant for adsorption (g mg^−1^ min^−1^) and *k*_2_ is the rate constant for the pseudo-second-order adsorption process.

**Fig. 9:**
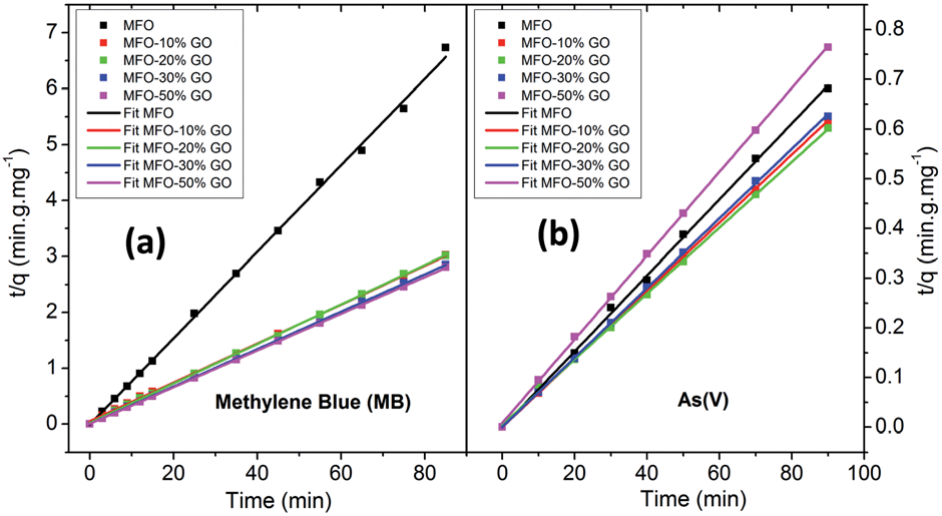
Pseudo-second-order kinetics plots for adsorption kinetics of different adsorbents for both (a) methylene blue (MB) dye and (b) arsenic(v) ions.

In order to fit our experimental data, we used both pseudo-first-order and pseudo-second-order kinetic models. The results that were obtained revealed that the pseudo-second-order kinetic model provided good fits for all the studied samples. Fig. 9 shows the fitted results for different adsorbent samples. The linear plots of *t*/*q*_e_*versus* time show good agreement between the experimental data and calculated values. The correlation coefficient (*R*^2^) for the pseudo-second-order model had high values of ∼99%. This result indicates that the adsorption process for samples of the MFO-GO nanocomposites closely corresponds to the pseudo-second-order equation.

**Fig. 10:**
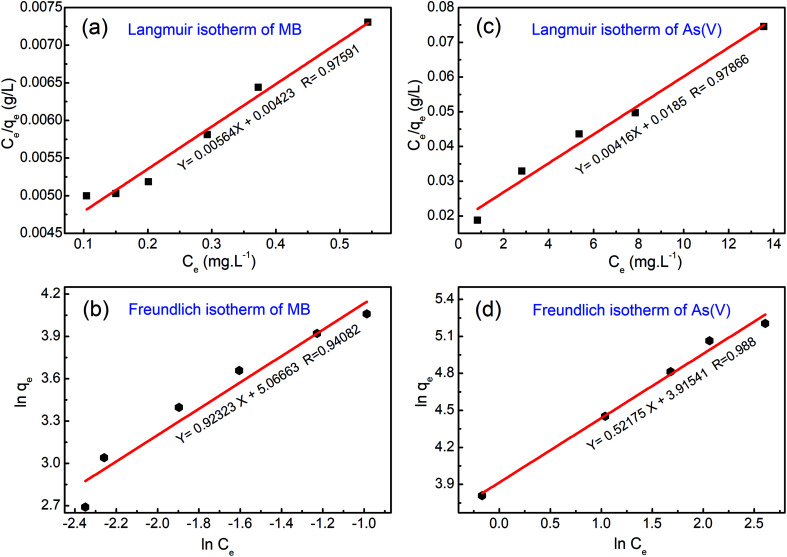
Experimental data fitted with the Langmuir and Freundlich isotherm models for (a and b) methylene blue (MB) dye and (c and d) arsenic(v) ions.

It is emphasized that the contact time is an important parameter for the determination of adsorption behavior over time, such as the removal efficiency and saturation adsorption time. Besides, the initial concentration of pollutants is also a key factor that influences the adsorption capacity of adsorbent materials. In the next experiments, we investigated the adsorption behavior of the adsorbents as a function of the initial concentrations of MB dye and arsenic(v). Among the studied samples, we selected a sample of the MFO-20 wt% GO nanocomposite to quantify the maximum adsorption capacity of the nanocomposite adsorbents because of its high removal efficiency and short saturation adsorption time for both MB dye and arsenic(v) ions, as mentioned above. The adsorption data were also fitted to two well-known models, namely, the Langmuir and Freundlich isotherms (see Fig. 10). Analysis of the isotherm data is necessary to develop a model that accurately represents the adsorption results. The Langmuir isotherm model assumes that the adsorbent surface only comprises a surface monolayer and adsorption occurs homogeneously. The Langmuir isotherm is expressed as follows:6
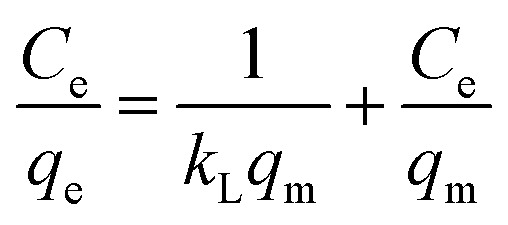
where *q*_e_ and *q*_m_ are the amounts of arsenic(v) or MB dye (mg g^−1^) adsorbed on the adsorbent at the equilibrium and maximum adsorption capacities, respectively, *C*_e_ is the equilibrium concentration of arsenic(v) or MB dye in the aqueous solution (mg L^−1^), and *k*_L_ is the Langmuir binding constant (L mg^−1^).

In the case of MB dye (see Fig. 10(a)), plotting *C*_e_/*q*_e_ against *C*_e_ gives a straight line of which the slope and intercept are 1/*q*_m_ and 1/(*k*_L_*q*_m_), respectively. From the slope and intercept, the values of *q*_m_ and *k*_L_ could be estimated to be 177.3 mg g^−1^ and 1.33 L mg^−1^, respectively, whereas the correlation coefficient (*R*^2^) is about 0.97591. Similarly, in the case of arsenic(v) (see Fig. 10(c)), the values of *q*_m_ and *k*_L_ could be estimated to be 240.4 mg g^−1^ and 0.00416 L mg^−1^, respectively, whereas the correlation coefficient (*R*^2^) is about 0.97866.

The Freundlich isotherm model describes the multilayer adsorption of an adsorbate on a heterogeneous adsorbent surface. The Freundlich isotherm is represented by the following equation:7
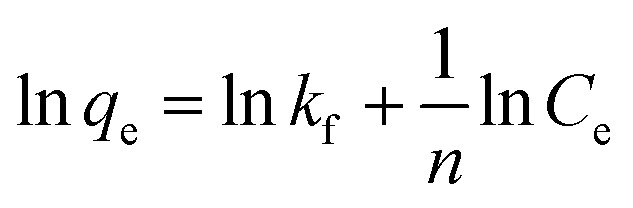
where *C*_e_ is the equilibrium concentration of arsenic(v) or MB dye in the solution (mg L^−1^), *q*_e_ is the amount of arsenic(v) or MB dye (mg g^−1^) adsorbed on the adsorbent at the equilibrium adsorption capacity, *k*_f_ is the Freundlich binding constant (L mg^−1^) and 1/*n* is a constant related to the surface heterogeneity. In the case of MB dye (see Fig. 10(b)), plotting ln (*q*_e_) against ln (*C*_e_) gives a straight line of which the slope and intercept are 1/*n* and ln (*k*_f_), respectively. The correlation coefficient (*R*^2^) is about 0.94082. Similarly, in the case of arsenic(v) (see Fig. 10(d)), the *R*^2^ value is about 0.988.

Batch experiments were carried out to quantify the adsorption kinetics and adsorption capacities of samples of the MFO-GO nanocomposites. The data that were obtained suggest that the Langmuir isotherm model and pseudo-second-order equation were more suitable for describing the adsorption process of MB dye on the MFO-GO nanocomposites, whereas the adsorption behavior of arsenic(v) displayed better agreement with the Freundlich isotherm model and pseudo-second-order equation. The maximum adsorption capacities of the MFO-GO nanocomposites reached high values of 177.3 mg g^−1^ for MB dye and 240.4 mg g^−1^ for arsenic(v) ions. A summary of the adsorption performance of different nanoadsorbents is given in Table 1. This revealed that our MFO-GO nanocomposite with an optimized GO content (∼20 wt%) exhibited some promising advantages, namely, greater removal efficiency, a higher maximum adsorption capacity and a shorter adsorption time in comparison with other reported adsorbents.^18–25^ This can provide new opportunities to further enhance the removal performance of different pollutants from aqueous solutions by using a functional magnetic MFO-GO composite system. Furthermore, the regenerability and reusability of this adsorbent also exhibited high performance, in that >98% of the adsorbed arsenic(v) ions were released from the MFO-GO adsorbent. The MFO-GO nanocomposites can be effectively reused for five cycles.^13^

**Table 1:** Comparison of adsorption performance of different nanoadsorbents

Adsorbent	Pollutant removal	Synthesis method	pH or *T* (K)	Removal efficiency (%)	Maximum adsorption capacity (mg g^−1^)	Saturation adsorption time (min)	Ref.
MnFe_2_O_4_-graphene	MB dye, UV light irradiation	Solvothermal, 180 °C for 20 h	25 °C	100	—	300	10
MnFe_2_O_4_ nanoparticles	MB dye, UV light irradiation	Solvothermal, 180 °C for 20 h	25 °C	80	—	300	10
rGO/MnFe_2_O_4_	MB dye, MB decomposition with H_2_O_2_	Hydrothermal, 150 °C for 15 h	Room temp	78	—	130	11
TiO_2_-graphene oxide (10 wt%)	MB dye, adsorption-enhanced photocatalysis	One-step colloidal blending	Room temp	90	37.26	75	18
TiO_2_ (P25)	MB dye, adsorption-enhanced photocatalysis	One-step colloidal blending	Room temp	50	5.01	75	18
Fe_3_O_4_-chitosan-GO	MB dye adsorption	Co-precipitation, 60 °C for 1 h	Room temp, pH = 7	—	95.16	80	20
MnFe_2_O_4_-GO nanocomposite	MB dye adsorption	Co-precipitation, 80 °C for 1 h	Room temp, pH = 7	95	177.3	20	This work
GO-MnFe_2_O_4_ nanohybrids	As(iii) adsorption	Co-precipitation, 80 °C for 5 min	298 K	96	146	40	12
	As(v) adsorption	Co-precipitation, 80 °C for 5 min	298 K	99.5	207	40	12
MnFe_2_O_4_ nanoparticles	As(iii) adsorption	Co-precipitation, 80 °C for 5 min	298 K	94	97	40	12
	As(v) adsorption	Co-precipitation, 80 °C for 5 min	298 K	98	136	40	12
Fe_3_O_4_-GO composite	As(v) adsorption	Co-precipitation, 80 °C	Room temp	88	59.6	240	15
Mesoporous MnFe_2_O_4_ nanoparticles	As(v) adsorption	Co-precipitation, 80 °C for 6 h	Room temp	—	68.25	400	22
NiFe_2_O_4_-GO composite	As(iii) adsorption	Co-precipitation, 80 °C for 45 min	Room temp	53	59.52	150	23
	As(v) adsorption	Co-precipitation, 80 °C for 45 min	Room temp	99.7	81.30	150	23
Iron-manganese binary oxide (FeMnO_*x*_)-rGO	As(iii) adsorption	Co-precipitation, 60 °C for 2 h	Room temp	—	22.17	300	24
	As(v) adsorption	Co-precipitation, 60 °C for 2 h	Room temp	—	22.05	300	24
Fe_3_O_4_-GO composite	As(iii) adsorption	Co-precipitation, 80 °C for 1.5 h	Room temp	—	54.18	250	25
	As(v) adsorption	Co-precipitation, 80 °C for 1.5 h	Room temp	—	26.76	250	25
MnFe_2_O_4_-GO nanocomposite	As(v) adsorption	Co-precipitation, 80 °C for 1 h	Room temp	99.9	240.4	20	This work

#### c. Proposed adsorption mechanism of MB and arsenic(v)

**Fig. 11:**
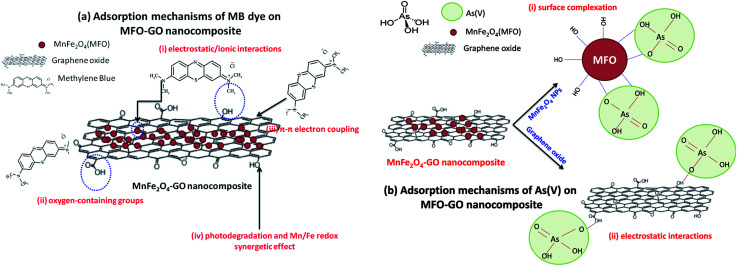
Proposed adsorption mechanisms of (a) methylene blue (MB) dye and (b) arsenic(v) ions on GO-MnFe_2_O_4_ nanocomposites.

Several mechanisms have been reported to explain the adsorption of MB dye and arsenic(v).^18–25^ Firstly, the possible mechanisms of the adsorption of MB dye in a neutral solution can be understood in terms of four main factors (see Fig. 11(a)), namely: (i) electrostatic/ionic interactions. The first reason is attributed to the electrostatic attraction of positively charged MB molecules and negatively charged –OH groups on the surfaces of both GO nanosheets and MFO nanoparticles; (ii) oxygen-containing groups. The second reason relates to oxygen-containing groups such as carboxyl (–COOH), epoxy (C–O), and hydroxyl (–OH) on the basal planes and edges of GO sheets. In this case, the functional groups play the role of active binding sites for the adsorption of MB molecules; (iii) π–π conjugation. The third reason is associated with π–π electron donor–acceptor interactions with graphene surfaces. Because MB contains C=C double bonds and π electrons, these π electrons can easily interact with the π electrons of benzene rings on GO surfaces *via* π–π electron coupling; and (iv) photodegradation and synergetic effects. This reason comprises the synergetic effect of adsorption and photocatalysis, which results in the efficient decomposition of MB dye. In addition, an Mn/Fe redox synergetic mechanism is important in MFO catalysts for MB degradation.

These reported explanations indicate the important role of graphene oxide in the adsorption of MB dye, in which the GO nanosheets act as key components in the three mechanisms of electrostatic/ionic interactions, oxygen-containing groups and π–π conjugation, whereas the MFO nanoparticles mainly contribute to the photodegradation mechanism and make a partial contribution to electrostatic interactions owing to residual –OH groups on the surface of the MFO nanoparticles.^18–20^ With this mechanism in mind, when we increase the GO concentration a much higher density of oxygen-containing functional groups such as carboxyl, epoxy and hydroxyl groups can be achieved, which leads to more ionic/electrostatic interactions and π–π electron couplings, and therefore greater adsorption activity will be achieved. This agrees well with our adsorption data, in which with an increase in the GO concentration the adsorption efficiency for MB dye of the MFO-GO nanocomposites was enhanced. It is noted that the high adsorption performance for MB dye of the MFO-GO nanocomposites is not only attributed to the synergetic effect of GO and MFO nanoparticles but is also related to the significant role of the GO content in the samples. Therefore, the increase and control of the GO content in the composite system with MFO nanoparticles play an important role in optimizing their adsorption ability for MB dye.

Next, some other explanations have been proposed to explain the adsorption mechanisms of arsenic(v) ions in solutions with various pH values.^21,22^ By correlating the zeta potential with the pH, it was reported that the pH of the solution had a great effect on the surface characteristics of the adsorbent and adsorbate. Hu *et al.*^22^ studied the behavior and mechanism of the adsorption of different arsenic species on mesoporous MnFe_2_O_4_ magnetic nanoparticles, and the authors revealed that the adsorption capacity of MnFe_2_O_4_ for arsenic species decreased with an increase in pH from 2 to 12 and reached a maximum around a pH of 2. In addition, it was found that a decrease in the potential of zero charge confirmed that the adsorption of different species of arsenic was mainly based on negatively charged inner-sphere complexes between arsenic species and MnFe_2_O_4_ nanoparticles.^22–25^ In the pH range of 2–3 arsenic species existed in two main forms, namely, H_3_AsO_4_^0^ and H_2_AsO_4_^−^; when the pH was in the range of 3–6 H_2_AsO_4_^−^ and HAsO_4_^2−^ were the predominant species; whereas at high pH values in the range of 6–12, HAsO_4_^2−^ and AsO_4_^3−^ were the predominant species.^22^ In the present case, the adsorption experiments were conducted at low pH values (1–2). Therefore, mechanisms for explaining the adsorption of arsenic(v) on MFO-GO nanocomposites were discussed in terms of arsenic(v) species in the form of H_2_AsO_4_^−^. Currently, the enhanced adsorption of H_2_AsO_4_^−^ on MFO-GO nanocomposites can be understood *via* two factors (see Fig. 11(b)): (i) surface complexation. This is associated with the adsorption of H_2_AsO_4_^−^ ions on the surface of MFO nanoparticles *via* the formation of an inner-sphere complex between arsenic acid moieties and surface metal centers with As–O–M (M = Fe or Mn) linkages. During the adsorption process, As–O(H) groups from H_2_AsO_4_^−^ species can undergo ligand exchange reactions with OH groups from the MnFe_2_O_4_ surface to form an inner-sphere complex; and (ii) electrostatic interactions. This adsorption mechanism is attributed to a chelation reaction between oxygen-containing functional groups (*e.g.*, carboxyl (–COOH) and hydroxyl (–OH)) on GO nanosheets and arsenic(v) ions. In conditions of low pH the number of H ions in the solution increases, and –OH and –COOH groups become positively charged –OH_2_^+^ and –COOH_2_^+^ groups, which is beneficial for the adsorption of negatively charged arsenic(v) ions (H_2_AsO_4_^−^). It was noted that in the electrostatic interaction mechanism the role of GO nanosheets in the adsorption of arsenic(v) was predominant, which was different from the previous surface complexation mechanism in which the role of MFO nanoparticles was predominant.

These abovementioned mechanisms suggest the important role of the GO content in improving electrostatic interactions between functional groups and arsenic(v) species and therefore enhancing the adsorption capacity. This shows good agreement with our experimental data, in which when we increased the GO concentration the removal efficiency of the MFO-GO nanocomposites was improved, which was mainly due to an increase in electrostatic interactions between oxygen-containing groups and H_2_AsO_4_^−^ ions. However, with a further increase in the GO concentration (MFO-50 wt% GO), the removal efficiency for arsenic(v) ions of the MFO-GO nanocomposite was drastically reduced, which was due to a reduction in surface interactions of MFO nanoparticles with arsenic(v) ions, as the MFO nanoparticles were wrapped by a large number of GO sheets.

## 4. Conclusions

In this study, MFO-GO nanocomposites with MFO particles with a diameter of ∼20 nm were synthesized using a modified co-precipitation method. We used the prepared MFO-GO nanocomposites for studies of the adsorption of organic and heavy metal pollutants, *i.e.*, MB dye and arsenic(v), from aqueous solutions, and their adsorption activities were compared with those of pure MFO nanoparticles and GO nanosheets. By fitting with the Langmuir model, the maximum adsorption capacities (*q*_m_) of the MFO-GO nanocomposites were calculated to be about 177.3 mg g^−1^ for MB dye and 240.4 mg g^−1^ for arsenic(v) ions. The effects of the GO concentration on the adsorption activities and mechanisms of both MB dye and arsenic(v) ions were studied in detail. The results that were obtained revealed the beneficial role of the graphene oxide content in the adsorption mechanisms of both MB dye and arsenic(v) ions, in which the enhanced adsorption mechanism of MB dye in a neutral aqueous solution was mainly attributed to (i) an increase in electrostatic/ionic interactions between the positively charged MB dye and negatively charged oxygen-containing functional groups at the edges or on the surfaces of GO nanosheets, as well as (ii) a large increase in the number of active binding sites as the GO content increased and (iii) the formation of π–π interactions between dye molecules and aromatic carbon rings, whereas the adsorption mechanism of arsenic(v) ions in an acidic aqueous solution was associated with (iv) an enhancement of electrostatic interactions due to chelation reactions between positively charged –OH_2_^+^ and –COOH_2_^+^ functional groups on GO nanosheets and negatively charged arsenic(v) ions (H_2_AsO_4_^−^) and (v) synergetic effects of both MFO nanoparticles and GO nanosheets.

## Conflicts of interest

There are no conflicts to declare.

## Supplementary Material
